# The association between food preferences, eating behavior, and body weight among female university students in the United Arab Emirates

**DOI:** 10.3389/fpubh.2024.1395338

**Published:** 2024-07-23

**Authors:** Haleama Al Sabbah, Abir Ajab, Leila Cheikh Ismail, Ayesha Al Dhaheri, Sharifa Alblooshi, Siham Atari, Stephanny Vicuna Polo, Malak Amro, Radwan Qasrawi

**Affiliations:** ^1^Department of Public Health, College of Health Sciences, Abu Dhabi University, Abu Dhabi, United Arab Emirates; ^2^Department of Clinical Nutrition and Dietetics, College of Health Sciences, University of Sharjah, Sharjah, United Arab Emirates; ^3^Nuffield Department of Women’s and Reproductive Health, University of Oxford, Oxford, United Kingdom; ^4^Department of Nutrition and Health, College of Medicine and Health Sciences, United Arab Emirates University, Al Ain, United Arab Emirates; ^5^Department of Health Sciences, College of Natural and Health Sciences, Zayed University, Dubai, United Arab Emirates; ^6^Department of Computer Science, Al-Quds University, Jerusalem, Palestine; ^7^Department of Computer Engineering, Istinye University, Istanbul, Türkiye

**Keywords:** obesity, food preferences, eating habits, lifestyle, fast food consumption patterns

## Abstract

**Introduction:**

This cross-sectional study investigated the associations between lifestyle, eating habits, food preferences, consumption patterns, and obesity among female university students in the United Arab Emirates (UAE).

**Methods:**

Approximately 4,728 participants, including both Emirati and Non-Emirati students (International Students). Data collection involved face-to-face interviews and anthropometric measurements, showing an interrelated relationship between food preferences and obesity among female university students.

**Results:**

While sociodemographic factors and lifestyle habits contribute to obesity, this study uniquely focuses on the role of food preferences and food consumption patterns in body weight status. The findings reveal a significant correlation between the intake of high-sugar beverages–such as milk, juices, soft drinks, and energy drinks–and an increased risk of overweight and obesity among both Emirati and Non-Emirati populations. Notably, milk consumption was particularly associated with obesity in non-Emirati populations (*F* = 88.1, *p* < 0.001) and with overweight status in Non-Emiratis (*F* = 7.73, *p* < 0.05). The consumption of juices and soft drinks was linked to obesity. Additionally, a significant preference for fruits and vegetables among overweight and obese students was observed, indicating a trend toward healthier food choices. However, there was also a clear preference for high-calorie, low-nutrient foods such as processed meats, sweets, and salty snacks. Fast food items like burgers, fried chicken, fries, pizza, shawarma, chips, and noodles were significantly correlated with increased body weight status, especially shawarma, which showed a notably high correlation with both obesity and overweight statuses (*F*-values of 38.3 and 91.11, respectively).

**Conclusion:**

The study indicated that food choices shape weight-related outcomes is important for designing effective strategies to promote healthier dietary patterns.

## 1 Introduction

Obesity has become a significant health concern globally, especially among university students (1). This population experiences substantial changes in their eating habits and food preferences as they navigate over their college years (2–4). During their university years, students experience significant changes, not only academically but also in their food choices and how they eat (5). The rise in obesity can be attributed to various factors, including a shift toward Western diets characterized by the consumption of processed foods, sugary drinks, and fast food (6, 7). Factors such as higher disposable incomes, busy lifestyles, and extensive promotion of unhealthy foods drive this dietary shift. Moreover, less physical activity due to sedentary lifestyles contributes to this issue (6, 8, 9).

The relationship between food preferences and obesity is a critical area of research in nutrition and public health (10, 11). With the global increase in obesity, understanding how dietary patterns impact health has become essential (12). According to the WHO, high intakes of sodium, sugars, and lipids have been linked to the development of chronic diseases such as obesity, diabetes, and cardiovascular diseases (13). Hall et al. suggested that the principal reason for being overweight is that people consume more energy (kcal) than they expend (14). The study of Popkin et al. showed that high-calorie, processed foods, rich in processed grains, sugars, and unhealthy fats, are strongly linked to weight gain and obesity (15). In several recent decisive studies from the United States and Europe, it has been shown that high consumption of ultra-processed foods is causally linked to obesity (10, 16, 17). The accessibility and palatability of these foods frequently result in overconsumption and increased calorie intake. These changing food preferences significantly contribute to the problem of obesity among university students (7, 10). Studies have shown that the risk of obesity is higher during the university years, with approximately 36.1% of university students in the United States being overweight or obese (18, 19). This trend is consistent with global patterns, highlighting obesity as a pressing issue among young adults attending universities worldwide (20, 21). Furthermore, the influence of peers and social networks plays a significant role. University life typically involves communal dining and shared food choices, which can lead to peer pressure to conform to unhealthy eating habits, ultimately leading to higher calorie intake (22). Moreover, cultural and socioeconomic factors also significantly influence food preferences and obesity (23, 24).

This study investigates the associations between obesity, food preferences, and dietary patterns within the university student population in the United Arab Emirates (UAE). Which is a novel approach that provides a specific focus on female university students in the UAE, an underrepresented demographic in existing literature. It uniquely includes both Emirati and non-Emirati students, enabling a comparative analysis of food preferences and obesity rates. The study provides a detailed examination of specific food items and their direct associations with body weight, such as the correlation between shawarma consumption and increased obesity. Additionally, it integrates a wide range of sociodemographic and lifestyle factors, offering a comprehensive view of the complex influences on body weight in this population.

The research aims to explore how factors related to food preferences and consumption patterns influence choices of the food and eating habits of participating students, thereby enhancing our comprehension of the obesity epidemic within this specific demographic. The primary focus of this study is to explore the associations between body weight, food composition, and dietary habits among these participants. It involves a detailed assessment of eating habits, encompassing considerations of nutrient intake, perceptions of taste and texture, and the speed at which food is consumed. Furthermore, the research seeks to gage fundamental taste preferences, including sweetness, saltiness, and fat, in commonly consumed foods within the UAE. By addressing these aspects comprehensively, the study contributes valuable insights to the broader understanding of obesity and its implications among female university students in the UAE.

### 1.1 Study design and settings

A cross-sectional study was conducted on female university students, including Emirati and Non-Emirati residents in the UAE. The present study followed the principles outlined in the Declaration of Helsinki, with all procedures involving human subjects receiving approval from the Zayed University Ethical Committee (No. ZU20_163_F).

The inclusion criteria for this study required participants to be female students aged between 18 and 30 years, currently enrolled at either Zayed University or the University of Sharjah. Participants needed to provide informed consent to participate and could include both Emirati and non-Emirati students. Additionally, participants had to be generally healthy without any chronic illnesses that could affect their dietary habits or body weight.

The exclusion criteria specified that male students and individuals under 18 or over 30 years of age were not eligible. Students not enrolled at Zayed University or the University of Sharjah were also excluded. Participants who did not provide informed consent, those with chronic illnesses or medical conditions significantly affecting dietary habits or body weight, and pregnant students were not included in the study. Furthermore, individuals with incomplete or missing data on key variables necessary for the study analysis were excluded.

### 1.2 Population and sampling

Participants for this study were recruited from two public universities in the UAE (Zayed University and University of Sharjah), where the predominant nationality is Emirati. Utilizing convenience sampling, recruitment strategies such as classroom announcements and advertisements were employed to identify potential participants easily accessible within the university setting. The study aimed to have a random sample of 680 participants, with a balanced representation of Emirati and Non-Emirati individuals, employing stratified sampling techniques to ensure proportional inclusion from both demographics. The dataset was balanced by nationality, age group, and BMI categories, through the utilization of the Synthetic Minority Over-sampling (SMOTE) technique (25, 26). The implementation of SMOTE involved creating synthetic samples by interpolating between instances of the less represented class. This technique generated new samples by selecting pairs of neighboring minority class instances and producing new data points along the line connecting them. This method adheres to the O = 2 k heuristic for estimating sample size, where ‘k’ represents the number of variables, ensuring a robust subject pool for effective analysis (26). While some literature recommends larger sample sizes, up to 60 k or 70 k per variable for greater statistical power, our adjusted sample size adheres to these standards, balancing statistical robustness and practical feasibility for identifying distinct groups. Consequently, this approach expanded our sample size to 4,728 participants, ensuring a substantial and robust pool for our analysis.

### 1.3 Measurements

The study employed a questionnaire to gather detailed information regarding how food preferences, eating habits, and other factors interrelate with obesity among students at public universities in the UAE, encompassing a diverse demographic of Emirati and Non-Emirati individuals across various body mass index (BMI) categories. The study mainly looked at sex main things.

#### 1.3.1 Sociodemographic variables

Includes the participants’ age, education level, place of residence, family income and marital status.

#### 1.3.2 Lifestyle

Including sleeping hours (≤5 h, 6–8 Hours, and > 8 h) (27). Physical activity was measured as moderate physically active (up to 150 min per week), or intensively active (up to 300 min per week) (28).

#### 1.3.3 Body mass index

Weight and height were measured using calibrated scales and stadiometers. Body weight was measured to the nearest 0.1 kg, with participants wearing minimal clothing and no shoes. BMI was calculated by dividing weight in kilograms by the square of height in meters (kg/m^2^). BMI categories were defined as follows: underweight (BMI < 18.5 kg/m^2^), normal weight (BMI = 18.5–24.9 kg/m^2^), overweight (BMI = 25–29.9 kg/m^2^), and obese (BMI ≥ 30 kg/m^2^) (29).

#### 1.3.4 Understanding dietary patterns

The study investigated the dietary habits using a validated Food Frequency Questionnaire (FFQ) and the food consumption pattern scale (30). This questionnaire provided valuable insights into the dietary patterns and choices of the participants (31). The reliability of the FFQ scale was assessed using Cronbach’s Alpha. The scale, composed of 13 items, yielded a Cronbach’s Alpha coefficient of 0.78. This indicates a very good internal consistency. Moreover, the scales include items such as Milk, Juice, Soft drinks, Energy drinks, Coffee, and Tea. The scales are categorized into four categories: Never, Daily, Weekly, and Monthly, alongside a measure of daily consumption in cups. For milk, participants report their intake frequency using the given scale and also specify the number of cups they consume each day.

#### 1.3.5 The food preferences scale

The study utilized a comprehensive food preferences scale, employing a three-level system (“Do not Like,” “Like,” and “Like A Lot”) (32), to assess participants’ preferences across various food categories. This scale allowed participants to express their liking for each food item, providing valuable insights into their dietary inclinations. For instance, preferences for fruits, vegetables, legumes, nuts, and different types of unprocessed meats (such as fish, poultry, and red meat) were evaluated to understand participants’ inclinations toward healthier food options. Similarly, assessments were made regarding preferences for sweets, salty snacks, bread (including general and white bread), milk, dairy products, and a variety of beverages, including non-sugar and sugar-sweetened beverages, energy drinks, and regional drinks like Arabian sweet beverages. Furthermore, the scale was applied to assess preferences for processed meats and specific items such as tomatoes and yogurt, offering a detailed understanding of participants’ food choices. Additionally, the scale explored preferences for culturally specific items like cooked food (Sawani) and rice, providing insights into participants’ cultural dietary patterns. Furthermore, the reliability of the Food Preferences Scale was assessed using Cronbach’s Alpha. The scale, composed of 22 items, yielded a Cronbach’s Alpha coefficient of 0.89. This indicates excellent internal consistency.

#### 1.3.6 Fast-food consumption

Scale with five categories is designed to measure how often individuals eat certain fast foods. The categories are “Never,” “1–2 times a month,” “Less than 4 times a month,” “Once a week,” and “2–4 times a week.” This scale is applied to various fast-food items to measure their frequency in participants’ diets. For each item - burgers, fried chicken, fries, pizza, shawarma, chips, and noodles - participants select one of the five frequency options. “Never” indicates no consumption, “1–2 times a month” and “Less than 4 times a month” represent occasional consumption, “Once a week” suggests a regular weekly intake, and “2–4 times a week” reflects a more frequent consumption (33, 34). Moreover, the reliability of the Food Consumptions Scale was assessed using Cronbach’s Alpha. The scale, composed of 7 items, yielded a Cronbach’s Alpha coefficient of 0.82. This indicates a very good internal consistency.

### 1.4 Data analysis

Data obtained were analyzed using the Statistical Package for Social Sciences software (SPSS, Inc.) version 21.0. To investigate variations in the anthropometric characteristics of participants, analysis of variance (ANOVA) has been employed. The findings are presented as means along with standard deviations (SD). To gain insights into the links between obesity and food preferences as well as consumption patterns, we applied binary logistic regression. All reported *p* values resulted from two-sided tests and were assessed against a significance level of 5%. Statistical significance was acknowledged when p values were less than 0.05.

## 2 Results

### 2.1 Selected sociodemographic characteristics and weight status

Table 1 presents the descriptive analysis of the sociodemographic characteristics and weight status. The age distribution is divided equally across three age groups (18–19, 20–21, and 22–23 years), with each group accounting for 33.3% of both Emirati and Non-Emirati populations. The majority (96.7%) of the study participants are living with their family (97.1%). While about 91.8% of Emirati students reported moderate- and high-income families, and 71.3 of Non-Emirati reported moderate- and high-income families.

**Table 1:** Descriptive analysis for selected sociodemographic characteristics and weight status.

Variable	Emirati (*n* = 2,364) *n* (%)	Non-Emirati (*n* = 2,364) *n* (%)	Total (*n* = 4,728) *n* (%)
Age (Years)			
18–19	788 (33.3)	788 (33.3)	1,576 (33.3)
20–21	788 (33.3)	788 (33.3)	1,576 (33.3)
22–23	788 (33.3)	788 (33.3)	1,576 (33.3)
Marital status			
Single	2,330 (98.6)	2,240 (94.8)	4,570 (96.7)
Married	34 (1.4)	124 (5.2)	158 (3.3)
Household sharing			
Live with family	2,321 (98.2)	2,269 (96)	4,590 (97.1)
Live alone or with roommates	43 (1.8)	95 (4)	138 (2.9)
Family income			
Low	195 (8.2)	678 (28.7)	873 (18.5)
Moderate	1,503 (63.6)	1,310 (55.4)	2,813 (59.5)
High	666 (28.2)	376 (15.9)	1,042 (22)

The univariate analysis in Table 2 reported distinct associations between selected sociodemographic variables and weight status categories. Age is significantly associated only with obesity (*F* = 23.3, *p* < 0.001). Marital status shows a strong association across all weight status categories, with the most significant impact observed in obesity (*F* = 73, *p* < 0.001), followed by underweight (*F* = 11.15, *p* < 0.001), and least in the overweight category (*F* = 4.5, *p* = 0.035). Household sharing was associated with underweight status (*F* = 36.8, *p* < 0.001), while this association is not observed in overweight or obese categories. Nationality’s impact is only evident in the obese participants (*F* = 22.8, *p* < 0.001). Lastly, family income is significantly associated with overweight (*F* = 7.7, *p* = 0.005) and highly significant associated with obese (*F* = 60.1, *p* < 0.001) categories.

**Table 2:** Univariate analysis of selected sociodemographic characteristics and weight status.

Sociodemographic	Underweight (*n* = 1,182) *F*-value	Overweight (*n* = 1,182) *F*-value	Obese (*n* = 1,182) *F*-value
Age (Years)	1.25	0.6	23.3**
Marital status	11.15**	4.5*	73**
Household sharing	36.8**	1.6	2.9
Nationality	0.08	0.8	22.8**
Family income	1.25	7.7*	60.1**

*Statistically significant: *P* value < 0.05, **Statistically highly significant: *p* value < 0.001.

### 2.2 Eating habits, food preferences and food consumption patterns

Table 3’s univariate analysis examines the relationship between lifestyle variables (smoking, physical activity, sleeping hours, eating habits, and water consumption) and weight status (underweight, overweight, obese) among Emirati and Non-Emirati groups. Smoking is linked to underweight in Non-Emiratis (*F* = 26.84, *p* < 0.001), and linked to obesity among Emiratis (*F* = 20.78, *p* < 0.001), and to overweight among non- Emiratis (*F* = 11.5, *p* < 0.001). Furthermore, physical activity correlates with underweight in Non-Emiratis (*F* = 38.02, *p* < 0.001) and with obesity and overweight in both groups. Sleeping hours are associated with underweight and obesity across both groups.

**Table 3:** Univariate analysis of lifestyle variables and weight status.

	Total (*n* = 4,728)	Underweight *F*-value		Overweight *F* -value		Obese *F*-value	
Lifestyle Variable	*n* (%)	Emirate *n* = 591	Non-emirate *n* = 591	Emirate *n* = 591	Non-emirate *n* = 591	Emirate *n* = 591	Non-emirate *n* = 591
Smoking	Yes (6.1)	2.05	26.84**	0.75	11.5**	20.78**	3.19
	No (93.9)						
Physical Activity	Yes (69.6)	0.46	38.02**	5.66*	38.17**	4.95*	50.85**
	No (30.4)						
Sleeping Hours	8+ (69)	37.08**	4.18*	3.72	1.98	17.67**	41.77**
	<5 (31)						
Lunch Frequency	Yes (29.9)	2.8	0.02	1.68	8.59*	23.05**	1.3
	No (70.1)						
Dinner Frequency	Yes (60.6)	3.82	46.08**	2.57	0.1	6.29*	0.48
	No (39.4)						
Breakfast Weekdays	Yes (59)	26.65**	3.29	0.12	0.76	6.05*	12.96**
	No (41)						
Breakfast Weekend	Yes (70.7)	13**	3.2	0.38	0.28	2.05	8.48*
	No (29.3)						
Eating Quantity	Normal (71.4)	0.09	23.97**	22.21**	23.35**	77.97**	3.23
	A lot (28.6)						
Eating Speed	Normal (59.9)	44.7**	2.23	0.01	0.04	16.3**	16.92**
	Quick (40.1)						
Water Drink	Normal (63.4)	32.28**	9.68*	2.11	22.91**	29.43**	17.04**
	Low (36.6)						

*Statistically significant: *P* value < 0.05; **Statistically highly significant: *P* value < 0.001.

Eating habits vary with weight status; with lunch and dinner frequencies showing mixed associations with weight categories. Specifically, lunch is associated with obesity in Emiratis. (*F* = 23.05, *p* < 0.001), and dinner with underweight in Non-Emiratis (*F* = 46.08, *p* < 0.001) and obesity in Emiratis (*F* = 6.29, *p* = 0.012). Weekday breakfast frequency is linked to underweight in Emiratis (*F* = 26.65, *p* < 0.001) and obesity in both groups, while weekend breakfast shows associations with underweight in Emiratis (*F* = 13, *p* < 0.001) and obesity in Non-Emiratis (*F* = 8.48, *p* = 0.004). Eating quantity and speed have significant linked to obesity, emphasizing their role in weight management. Moreover, the eating quantity had strong association with overweight and obesity among Emiratis (*F* = 222.2, *F* = 77.97, *p* < 0.001) respectively. The eating speed of Emiratis (*F* = 44.7, *p* < 0.001) exhibit a statistically highly significant association with underweight status, and associated with obesity among both groups. Water intake is significantly related to underweight and obesity in both groups (*F* = 32.28, *p* < 0.001; *F* = 9.68, *p* < 0.002), and (*F* = 29.4, *p* < 0.001; *F* = 17.0, *p* < 0.001) respectively.

Table 4’s univariate analysis investigates the relationship between food consumption patterns and weight status (underweight, overweight, obese) among Emirati and Non-Emirati groups. Milk intake is linked to underweight in Emiratis (*F* = 44.23, *p* < 0.001) and to overweight in Non-Emiratis (*F* = 7.73, *p* = 0.006), with a significant correlation to obesity among the non-Emirate (*F* = 88.1, *p* < 0.001). Juice consumption affects both underweight and obesity across both groups and overweight across non-Emirate (*F* = 7.57, *p* = 0.006), indicating a varied impact across BMI categories. Soft drinks are associated with underweight (*F* = 19.61, *p* < 0.001) and overweight (*F* = 10.81, *p* < 0.001) in Emiratis, with a significant link to obesity in non-Emiratis (*F* = 6.6, *p* = 0.01). Energy drinks correlate with underweight (*F* = 75.19, *p* < 0.001), overweight (*F* = 6.15, *p* = 0.013) in Emiratis and with obesity in both populations. Coffee shows a higher significant relationship with underweight in Emiratis (*F* = 25.54, *p* < 0.001) compared to non-Emirate (*F* = 6.4, *p* = 0.012), and with overweight in Non-Emiratis (*F* = 14.15, *p* < 0.001). Caffeinated beverage intake is significantly related to underweight and overweight in Emiratis and have a significant association with obesity within the non-Emiratis (*F* = 12.1, *p* < 0.001). Tea consumption is notably associated only with obesity more in Non-Emiratis (*F* = 13.4, *p* < 0.001) than in Emiratis (*F* = 5.6, *p* = 0.018).

**Table 4:** Univariate analysis of selected beverages consumption patterns and weight status.

	Total (*n* = 4,728)	Underweight (*n* = 1,182) *F*-value		Overweight (*n* = 1,182) *F*-value		Obese (*n* = 1,182) *F*-value	
Food consumption	*n* (%)	Emirate	Non-Emirate	Emirate	Non-Emirate	Emirate	Non-Emirate
Milk	>1 cup (74.8)	44.23**	4.08*	0.95	7.73*	0.1	88.1**
	Never/ rarely (25.2)						
Juices	>1 cup (75.7)	13.64**	61.61**	1.11	7.57*	21.3**	23.5**
	Never/ rarely (24.3)						
Soft drink	>1 cup (76.8)	19.61**	1.75	10.81**	0.31	1.2	6.6*
	Never/ rarely (23.2)						
Energy drink	>1 cup (87)	75.19**	0.21	6.15*	0.45	31.2**	29.5**
	Never/ rarely (13)						
Coffee	>1 cup (20.7)	25.54**	6.4*	3.1	14.15**	1.6	3.7
	Never/ rarely (79.3)						
Caffeinated	>1 cup (81)	27.94**	0.62	10.79**	0.14	3.1	12.1**
	Never/ rarely (19)						
Tea	>1 cup (75.1)	1.54	0.67	1.39	0.67	5.6*	13.4**
	Never/ rarely (24.9)						

*Statistically significant: *P* value < 0.05; **Statistically highly significant: *P* value < 0.001.

In Table 5, liking fruits is notably linked to weight status among Non-Emiratis (*F* = 23.7, 37.9, 7.0) and to underweight and overweight among Emirati students (*F* = 5.7, 21.4). Disliking vegetables is associated with overweight and obesity (*F* = 21.8, 23.6). Not liking legumes and nuts corresponds to various weight statuses in Emiratis (*F* = 5.8, 9.0, 11.7 for legumes; *F* = 6.68, 6.7, 15.6 for nuts) and to obesity in Non-Emiratis (*F* = 10.9 for legumes; *F* = 9.5 for nuts). Not liking unprocessed fish, poultry, and meat is related to obesity in Non-Emiratis (*F* = 16.9, 20.8, 10.6) and to overweight and obesity in Emiratis.

**Table 5:** Univariate analysis of food preferences and weight status.

Food preferences	Total (*n* = 4,728)	Underweight (*n* = 1,182) *F*-value		Overweight (*n* = 1,182) *F*-value		Obese (*n* = 1,182) *F*-value	
		Emirate	Non-Emirate	Emirate	Non-Emirate	Emirate	Non-Emirate
Fruit	Like a lot (54.5)	5.73*	23.67**	21.37**	37.91**	0.44	7.04*
	Do not like (45.5)						
Vegetables	Like a lot (42.7)	7.62*	2.23	21.8**	14.65**	23.56**	5.83*
	Do not like (57.3)						
Legumes	Like a lot (23.1)	5.8*	0.1	8.98*	1.23	11.71**	10.9**
	Do not like (76.9)						
Nuts	Like a lot (32.7)	6.68*	17.41**	6.67*	0.1	15.6**	9.48*
	Do not like (67.3)						
Unprocessed fish	Like a lot (29.2)	17.36**	0.73	0.21	0.1	0.86	16.86**
	Do not like (70.8)						
Unprocessed poultry	Like a lot (44)	2.49	9.26*	6.88*	0.05	183.86**	20.77**
	Do not like (56)						
Unprocessed meat	Like a lot (31)	0.95	0.07	0.01	19.68**	4.32*	10.58**
	Do not like (69)						
Sweets	Like a lot (18)	23.84**	22.79**	12.8**	14.44**	15.86**	6.17*
	Do not like (82)						
Salty snacks	Like a lot (21)	7.17*	14.77**	90.77**	0.16	5.54*	9.74*
	Do not like (79)						
Bread	Like a lot (48.2)	4.48*	23.81**	0.1	0.87	0.18	8.77*
	Do not like (51.8)						
White bread	Like a lot (46.4)	7.59*	119.95**	1.64	0.1	1.37	13.72**
	Do not like (53.6)						
Milks	Like a lot (30.2)	20.27**	23.87**	6.94*	2.27	4.29*	8.99*
	Do not like (69.8)						
Dairy product	Like a lot (44.9)	1.43	18.91**	6.83*	9.26**	3.22	38.1**
	Do not like (55.1)						
Non-sugar	Like a lot (35)	0.18	0.13	7.21*	1.39	4.67*	28.56**
	Do not like (65)						
Sugar beverage	Like a lot (35.2)	26.66**	50.66**	21.61**	10.04**	0.02	12.48**
	Do not like (64.8)						
Energy drink	Like a lot (5.8)	18.47**	0.22	4.92*	0.03	4.24*	50.06**
	Do not like (94.2)						
Processed meat	Like a lot (30.9)	0.05	5.17*	8.07*	0.01	7.41*	64.56**
	Do not like (69.1)						
Arabian sweet	Like a lot (22.5)	15.66**	17.33**	0.61	9.01*	0.05	0.28
	Do not like (77.5)						
Tomato	Like a lot (14.5)	4.95*	9.62*	6.57*	18.13**	5.72*	8.07*
	Do not like (85.5)						
Yogurt	Like a lot (11.2)	15.61**	12.03**	0.47	0.03	26.94**	11.72**
	Do not like (88.8)						
cooked food (Sawani)	Like a lot (24.6)	2.66	6.91*	10.34**	19.23**	0.51	2.65
	Do not like (75.4)						
Rice based	Like a lot (34.8)	3.93**	3.33	17.48**	1.56	14.4**	25.11**
	Do not like (65.2)						

*Statistically significant: *P* value < 0.05; **Statistically highly significant: *P* value < 0.001.

Disliking sweets and salty snacks are linked to multiple weight statuses, with a significant relation to overweight in Emiratis (salty snacks: *F* = 90.8, *p* = 0.001) and underweight in both groups (sweets: *F* = 7.17, *p* = 0.008 for Emiratis; *F* = 14.77, *p* < 0.001 for non-Emiratis). Bread and white bread show links to underweight and obesity (Bread: *F* = 8.77, *p* = 0.003; White Bread: *F* = 13.72, *p* < 0.001 in non-Emiratis). Milk preferences relate to various weight statuses (*F* = 6.94, *p* = 0.009 for overweight in Emiratis). Dairy preferences are significantly linked to overweight and obesity, especially in Non-Emiratis (*F* = 38.1, *p* < 0.001). Non-sugar beverages correlate with obesity (*F* = 7.21, *p* = 0.007 in Emiratis). Dislike to sugary drinks is associated with underweight and obesity (*F* = 12.48, *p* < 0.001 in non-Emiratis). Energy drinks show a strong link to underweight in Emiratis (*F* = 18.47, *p* < 0.001) and obesity in Non-Emiratis (*F* = 50.06, *p* < 0.001). Arabian sweets, tomatoes, and cooked food (Sawani) exhibit significant associations across weight statuses (cooked food (Sawani): *F* = 6.91, *p* = 0.009 for overweight). Rice-based foods and processed meats are significantly linked to obesity and other weight statuses (processed meats: *F* = 5.17, *p* = 0.023 for underweight in Non-Emiratis; *F* = 8.07, *p* = 0.005 for overweight in Emiratis). Yogurt shows surprising correlations with both obesity and underweight in both groups.

Table 6’s univariate analysis reveals how fast-food consumption patterns correlate with weight statuses (underweight, overweight, obese) among Emirati and Non-Emirati groups. Burger intake is linked to the three weight statuses in both groups. Fried chicken consumption correlates with underweight in Emiratis (*F* = 4.06, *p* = 0.044) and obesity in both populations. Fries are associated with underweight among both groups and overweight in Emiratis (*F* = 11.78, *p* < 0.001). Pizza consumption is significantly linked to underweight in Emiratis (*F* = 5.1, *p* = 0.024), overweight in non-Emiratis (*F* = 5.16, *p* = 0.023) and obesity in both groups. Shawarma, Chips and Noodles intake significantly correlates with all mentioned weight status across Emirati and Non-Emirati groups.

**Table 6:** Univariate analysis of selected fast-food consumption patterns and weight status.

Fast Food	Total (*n* = 4,728)	Underweight *F*-value		Overweight *F*-value		Obese *F*-value	
		Emirate	Non-Emirate	Emirate	Non-Emirate	Emirate	Non-Emirate
Burger	Yes (19.2)	21.24**	11.67**	4.67*	18.55**	8.64*	8.64*
	No (80.8)						
Fried Chicken	Yes (35.9)	4.06*	0.85	3.29	3.36	16.98**	16.98**
	No (64.1)						
Fries	Yes (53.2)	4.5*	16.38**	11.78**	0.71	1.05	1.05
	No (46.8)						
Pizza	Yes (31.2)	5.1*	0.1	0.01	5.16*	13.36**	13.36**
	No (68.8)						
Shawarma	Yes (32.4)	67.97**	17.28**	91.11**	38.3**	26.48**	26.48**
	No (67.6)						
Chips	Yes (47.6)	50.62**	12.64**	8.2*	4.17*	26.77**	26.77**
	No (52.4)						
Noodles	Yes (61.0)	4.36*	28.8**	22.49**	37.81**	6.68*	6.68*
	No (39.0)						

*Statistically significant: *P* value < 0.05; **Statistically highly significant: *P* value < 0.001.

This analysis indicates that certain fast foods like shawarma and chips impact multiple weight statuses, while others such as burgers and fries show specific associations within the Emirati and Non-Emirati populations.

### 2.3 Regression analysis

Table 7’s binary logistic regression analysis evaluates the impact of lifestyle, eating habits, and food consumption on weight status (underweight, overweight, obesity) among participants. The analysis reveals that non-smokers are significantly more likely to be overweight OR = 1.26, 95% CI: (0.83–1.91). Students who engage in physical activity show a reduced likelihood of being overweight (OR = 0.71, *p* < 0.05) and obese (OR = 0.59, *p* < 0.001), suggesting that an active lifestyle may protect against these conditions. In terms of sleep, those getting less than or equal to 5 h have significantly higher odds of obesity (OR = 2.29, *p* < 0.001), highlighting the importance of adequate sleep in maintaining a healthy weight. In eating habits, a larger eating quantity significantly increases the odds of being overweight (OR = 1.53, *p* < 0.001) and obese (OR = 2.53, *p* < 0.001). Similarly, a quick eating speed is associated with a significant increase in the odds of obesity (OR = 2.08, *p* < 0.001). Regarding water consumption, a lower intake of water significantly increases the odds of being underweight (OR = 2.69, *p* < 0.001), while regular consumption of soft drinks is significantly associated with higher odds of underweight (OR = 1.61, *p* < 0.001), overweight (OR = 1.57, *p* < 0.001), and obesity (OR = 1.75, *p* < 0.001). This indicates that not only the type of beverage but also the quantity consumed can influence weight status. Furthermore, regular coffee consumption is significantly associated with higher odds of being underweight (OR = 1.68, *p* < 0.001) and overweight (OR = 1.36, *p* < 0.05). These significant variables highlight potential targets for interventions aimed at improving weight-related health outcomes.

**Table 7:** Binary logistic regression of weight status by lifestyle factors, eating habits, and beverage consumption patterns.

	Underweight	Overweight	Obesity
Lifestyle	OR 95% C.I	OR 95% C.I	OR 95% C.I
Smoking (No, Yes)	0.26 (0.11–0.58) **	1.26 (0.83–1.91)	0.61 (0.35–1.08)
Physical Activity (Yes, No)	1.01 (0.77–1.32)	0.71 (0.57–0.89) *	0.59 (0.43–0.8) **
Sleeping Hours (≤ 5Hours. 6- h)	1.2 (0.92–1.56)	0.83 (0.66–1.05)	2.29 (1.68–3.12) **
Eating habits			
Lunch (Regular, irregular)	0.73 (0.56–0.96) *	0.95 (0.76–1.19)	0.26 (0.19–0.37) **
Dinner (Regular, irregular)	0.62 (0.48–0.81) **	0.72 (0.58–0.9) *	1.01 (0.74–1.36)
Weekday Breakfast (Regular, irregular)	1.22 (0.92–1.61)	1.14 (0.91–1.43)	0.89 (0.65–1.23)
Weekend Breakfast (Regular, irregular)	1.14 (0.86–1.51)	0.92 (0.73–1.17)	1.17 (0.86–1.6)
Eating Quantity (Normal, A lot)	0.36 (0.27–0.49) **	1.53 (1.2–1.94) **	2.53 (1.88–3.41) **
Eating Speed (Normal, Quick)	0.56 (0.44–0.73) **	0.8 (0.65–0.99) *	2.08 (1.58–2.74) **
Food consumption pattern			
Water (Normal, Low)	2.69 (2.09–3.45) **	0.52 (0.41–0.65) **	0.91 (0.68–1.2)
Milk (Never, ≥ one Cup per day)	1 (0.77–1.31)	0.98 (0.77–1.24)	0.85 (0.63–1.16)
Juice (Never, ≥ one Cup per day)	0.45 (0.34–0.6) **	0.79 (0.63–1) *	1.58 (1.16–2.14) *
Soft Drink (Never, ≥ one Cup per day)	1.61 (1.22–2.15) **	1.57 (1.22–2.02) **	1.75 (1.25–2.46) **
Energy (Never, ≥ one Cup per day)	1 (0.69–1.44)	0.71 (0.49–1.02)	0.45 (0.29–0.7) **
Coffee (Never, ≥ one Cup per day)	1.68 (1.27–2.22) **	1.36 (1.09–1.69) *	1.13 (0.82–1.54)
Tea (Never, ≥ one Cup per day)	0.72 (0.56–0.91) *	1 (0.81–1.24)	1.15 (0.87–1.52)

*Statistically significant: *p* value < 0.05; **Statistically highly significant: *p* value < 0.001.

Table 8 indicates that not liking fruits is associated with a higher obesity risk (OR = 1.74, *p* < 0.05). A preference for vegetables increases the chances of being overweight (OR = 2.01, *p* < 0.001) and obesity (OR = 1.59, *p* < 0.05). Eating legumes is linked to greater risks of overweight (OR = 1.32, *p* < 0.05) and obesity (OR = 1.82, *p* < 0.001). Disliking unprocessed fish and poultry correlates with underweight (OR = 1.45, *p* < 0.05; OR = 1.61, *p* < 0.05), while preferring unprocessed meat ups the risk for overweight (OR = 1.98, *p* < 0.001) and obesity (OR = 1.78, *p* < 0.05). A liking for sweets is a factor for overweight (OR = 1.51, *p* < 0.05) and obesity (OR = 1.98, *p* < 0.001). White bread significantly relates to obesity (OR = 3.34, *p* < 0.001), as does disliking dairy with underweight (OR = 1.91, *p* < 0.001). Preferring non-sugar beverages is links to underweight (OR = 2.38, *p* < 0.001), and sugar beverages to obesity (OR = 2.24, *p* < 0.001). Energy drink consumption is associated with underweight (OR = 0.53, *p* < 0.05), while processed meat preference links to obesity (OR = 1.53, *p* < 0.05). Preference of Arabian sweets significantly influences obesity risk (OR = 2.61, *p* < 0.001). Regular yogurt intake is also a risk for obesity (OR = 1.93, *p* < 0.05), and cooked food for underweight (OR = 1.98, *p* < 0.001) and obesity (OR = 1.71, *p* < 0.05). Regarding fast food, burgers increase obesity risk (OR = 2.09), shawarma even more so (OR = 6.87, *p* < 0.001), fries linked to overweight (OR = 1.59, *p* < 0.05), and noodles linked to underweight (OR = 1.65, *p* < 0.05).

**Table 8:** Binary logistic regression of BMI groups (Underweight, overweight and obese) by food preferences and fast-food consumption.

	Underweight	Overweight	Obesity
Food Preferences ((Like, Do not Like))	OR (95% C.I)	OR (95% C.I)	OR (95% C.I)
Fruit	0.94 (0.7–1.25)	0.34 (0.26–0.45) **	1.74 (1.21–2.51) *
Vegetable	0.65 (0.48–0.88) *	2.01 (1.54–2.61) **	1.59 (1.13–2.25) *
Legumes	0.77 (0.55–1.06)	1.32 (1–1.73) *	1.82 (1.28–2.59) **
Nuts	1.22 (0.9–1.65)	0.91 (0.72–1.15)	1.2 (0.88–1.64)
Unprocessed fish	1.45 (1.04–2.02) *	1.09 (0.83–1.42)	1.9 (1.34–2.71) **
Unprocessed poultry	1.61 (1.15–2.25) *	0.56 (0.43–0.73) **	0.12 (0.08–0.17) **
Unprocessed meat	0.67 (0.48–0.93) *	1.98 (1.51–2.61) **	1.78 (1.26–2.51) **
Sweets	0.38 (0.26–0.54) **	1.51 (1.14–2) *	1.98 (1.39–2.84) **
Salty snake	1.06 (0.76–1.49)	1.66 (1.25–2.2) **	1.23 (0.87–1.72)
Bread	2.13 (1.62–2.79) **	0.71 (0.55–0.92) *	0.36 (0.25–0.53) **
White bread	0.26 (0.19–0.35) **	1.19 (0.92–1.54)	3.34 (2.33–4.78) **
Milk	0.7 (0.5–0.99) *	1.04 (0.79–1.36)	0.59 (0.42–0.81) **
Dairy product	1.91 (1.39–2.62) **	1.21 (0.95–1.54)	0.48 (0.34–0.67) **
Non-sugar beverages	2.38 (1.79–3.18) **	1.25 (0.96–1.63)	0.91 (0.65–1.28)
Sugar beverages	3.19 (2.36–4.32) **	1.28 (0.99–1.65)	2.24 (1.6–3.14) **
Energy drink	0.53 (0.32–0.89) *	0.41 (0.29–0.57) **	1.08 (0.67–1.73)
Processed meat	0.65 (0.48–0.88) *	1.07 (0.83–1.39)	1.53 (1.09–2.14) *
Arabian sweet	0.88 (0.64–1.19)	1.11 (0.85–1.44)	2.61 (1.82–3.72) **
Tomato	2.28 (1.54–3.38) **	1.31 (0.95–1.81)	2.81 (1.78–4.43) **
Yogurt	1.6 (1–2.57)	0.6 (0.44–0.83) *	1.93 (1.24–3) *
Cooked food	1.98 (1.34–2.93) **	1.04 (0.79–1.37)	1.71 (1.16–2.51) *
Rice	0.74 (0.55–1.01)	0.75 (0.58–0.96) *	0.24 (0.17–0.33) **
Fast food consumption pattern (Never, ≥ Once a week)			
Burge	0.31 (0.23–0.42) **	0.62 (0.48–0.8) **	2.09 (1.53–2.87)
Chicken	0.89 (0.65–1.22)	0.67 (0.51–0.88) *	0.57 (0.39–0.82)
Fries	0.63 (0.44–0.88) *	1.59 (1.15–2.21) *	1.32 (0.87–2.02)
Pizza	1.31 (0.95–1.83)	0.72 (0.54–0.98) *	1.14 (0.79–1.65)
Shawarma	0.63 (0.45–0.89) *	2.51 (1.87–3.37) **	6.87 (4.71–10.01)
Chips	1.75 (1.27–2.42) **	1.01 (0.75–1.37)	0.26 (0.18–0.39)
Noodles	1.65 (1.21–2.25) *	0.55 (0.42–0.73) **	1.2 (0.87–1.65)

*Statistically significant: *p* value < 0.05; **Statistically highly significant: *p* value < 0.001.

## 3 Discussion

The presented study, investigating the relationships between sociodemographic factors, lifestyle habits, dietary patterns, and BMI categories in Emirati and Non-Emirati populations, reveals a rich interaction of their influences on weight management.

Significant among our findings is the differential impact of age, marital status, and household composition on BMI. The noticeable effect of age on obesity, more so than on underweight, is likely attributable to age-related metabolic slowdown and reduced physical activity, which contribute to adiposity and metabolic dysfunction (35), potentially due to metabolic changes and decreased physical activity (21, 36). Marital status shows a notable association with all BMI categories, particularly obesity. This trend may reflect healthier lifestyle choices within marital partnerships, aligning with previous studies (8). Notably, living with family, interestingly, is linked to lower BMI, possibly due to shared healthy eating habits or cultural norms, though this relationship is not consistently observed in other studies (2, 4, 37). Moreover, the influence of nationality, notably apparent in the obese group, suggests possible differences in dietary habits, physical activity, or genetic factors between Emirati and Non-Emirati populations.

The study revealed that among smokers, there was a higher prevalence of obesity and overweight individuals, indicating a positive correlation between smoking and higher BMI categories. This aligns with existing research highlighting the metabolic effects of smoking, including changes in appetite and metabolism. Conversely, underweight individuals were less prevalent among smokers, suggesting a potentially protective effect of smoking against being underweight. However, it’s important to note that smoking is associated with numerous adverse health outcomes regardless of BMI status, showing the need for comprehensive smoking stopping interventions (38). The significant correlation observed between physical activities and underweight status among non-Emirati students, alongside overweight and obese categories in both groups, emphasizes the role of physical activity in managing weight across BMI ranges. Cultural, socioeconomic, and personal factors influence this connection, impacting foreign students’ ability to engage in physical activities and maintain healthy habits. Challenges such as diet changes, limited resources, and psychosocial stressors affect their engagement levels. Understanding these dynamics is crucial for promoting healthier lifestyles among diverse student populations (24, 39, 40).

Sleeping hours revealed interesting associations with BMI categories among university students, suggesting a relationship between sleep patterns and weight status. While the study did not establish a significant link between sleeping hours and obesity, it indicates the bidirectional influences between sleep duration, quality, and obesity risk (8). Breakfast frequency’s importance for underweight and obese groups emphasizes the role of regular breakfast consumption in weight management (41). Notably, eating quantity, speed, and water intake emerged as significant factors influencing multiple BMI categories, especially obesity, emphasizing their importance in weight management practices (4, 42).

The analysis revealed interrelated relationships between specific food items and BMI categories. Milk’s consumption associations with underweight in Emiratis and overweight in non-Emiratis necessitate further examination considering milk type, cultural preferences, and overall dietary patterns. Juice consumption demonstrated diverse impacts across all BMI categories, indicating the need to consider sugar content, frequency, and types of juices consumed. Soft and energy drinks displayed varied associations, emphasizing the importance of considering the type and frequency of consumption for a clearer understanding of their impact on weight status (40). Coffee and caffeinated beverages’ correlations with underweight and overweight suggesting a potential influence on appetite and metabolism, warranting further research (10, 18). Tea’s association with obesity in non-Emiratis may be linked to cultural preferences for sweetened tea beverages, aligning with studies suggesting the importance of considering tea type and added sugar content (42, 43).

The analysis investigated the food preferences, revealing further information on how they influence weight status. Fruit and vegetable consumption showed a significant relationship with underweight, overweight, and obesity, indicating the importance of considering overall intake patterns and portion sizes to understand their actual impact on weight status (9, 11). Legumes and nuts were associated with underweight in Emiratis and obesity in non-Emiratis, further importance of dietary patterns and weight management across different populations. The distinct associations of unprocessed fish and poultry with underweight and obesity, respectively, emphasize the importance of diversifying protein sources for better weight management. Sweets and salty snacks also exhibited varied patterns, indicating their complex influence on different BMI categories. Sugar beverages and energy drinks showed significant correlations with obesity, respectively, indicating their impacts on weight status. Similarly, processed meats, Arabian sweets, and yogurt reported specific associations with certain BMI categories, emphasizing the strong relationship between food preferences and weight management. For instance, both Emirati and Non-Emirati groups with underweight BMI showed a significant preference for sweets, suggesting strong relationships between sugar intake and weight management. Additionally, the association of fried chicken with underweight in Emiratis and obesity in both groups shows the importance of considering food preparation methods and their potential impact on weight. Interestingly, shawarma, a popular fast-food item in the region, showed a consistent association with overweight across both groups (3, 16).

### 3.1 Limitation

Despite its valuable insights, this study has important limitations. The cross-sectional design prevents establishing causality between food preferences, eating behaviors, and body weight. Self-reported data may introduce biases, affecting the accuracy of dietary and lifestyle information. Furthermore, the sample of female university students in the UAE may not represent the broader population, limiting generalizability. Cultural and dietary differences between Emirati and non-Emirati students are not fully accounted for, influencing food preferences and consumption patterns.

The study also lacks a deep exploration of behavioral and psychological factors, such as stress and emotional eating, which can affect eating behaviors and weight status. Future research should address these limitations to better understand the factors influencing body weight and obesity among university students in the UAE.

## 4 Conclusion

Our study provides valuable insights into the links between food preferences and fast-food consumption patterns with various BMI groups, including underweight, overweight, and obesity. Our study found that age played a significant role in obesity, possibly due to changing bodies and reduced physical activity as people age. Physical activity was consistently linked to maintaining a healthy weight, regardless of nationality. Sleep patterns, breakfast consumption, and meal habits also mattered. Eating slowly, staying hydrated, and regular meal times were associated with better weight management. Food preferences and fast-food consumption also played a role. Vegetables were beneficial, while sweets were not. Understanding these factors can help individuals make healthier choices to manage their weight effectively.

## Data availability statement

The raw data supporting the conclusions of this article will be made available by the authors, without undue reservation.

## Ethics statement

The studies involving humans were approved by Zayed University Ethical Committee (No. ZU20_163_F). The studies were conducted in accordance with the local legislation and institutional requirements. The participants provided their written informed consent to participate in this study.

## Author contributions

HS: Conceptualization, Data curation, Investigation, Methodology, Project administration, Supervision, Writing – review & editing. AA: Conceptualization, Methodology, Writing – review & editing. LI: Data curation, Methodology, Writing – review & editing. AD: Methodology, Writing – review & editing. ShA: Supervision, Writing – review & editing. SiA: Resources, Supervision, Writing – review & editing. SP: Data curation, Writing – review & editing. MA: Software, Supervision, Writing – review & editing. RQ: Conceptualization, Data curation, Formal analysis, Investigation, Methodology, Project administration, Writing – original draft.
